# Cell Adhesive Character of Phenylboronic Acid-Modified Insulin and Its Potential as Long-Acting Insulin

**DOI:** 10.3390/ph12030121

**Published:** 2019-08-19

**Authors:** Yui Ohno, Momoko Kawakami, Tomohiro Seki, Ryotaro Miki, Toshinobu Seki, Yuya Egawa

**Affiliations:** Faculty of Pharmacy and Pharmaceutical Sciences, Josai University, 1-1 Keyakidai, Sakado, Saitama 350-0295, Japan

**Keywords:** phenylboronic acid, cell adhesive, insulin, diabetes, long-acting insulin, red blood cell

## Abstract

Phenylboronic acid (PBA) derivatives have attracted substantial attention owing to their unique character of forming dynamic covalent bonds with polyol compounds. Recent studies have shown interactions between PBA and sugar chains on the cell surface; they have interesting applications for sensors and drug delivery systems. In this study, we prepared phenylboronic acid-modified insulin (PBA-Ins) to evaluate its glucose-lowering activity and cell adhesiveness. In the case of intravenous injection, PBA-Ins showed longer glucose-lowering activity than native insulin. We hypothesized that this prolonged effect was the result of the interaction between the PBA moiety and sugar chains on the cell surface. Red blood cells (RBCs) were used as a cell model, and we confirmed PBA-Ins’s affinity for RBCs, which induced RBC agglutination. Interestingly, using an alternative PBA-Ins administration route markedly changed its glucose-lowering activity. Unlike the intravenous injection of PBA-Ins, the subcutaneous injection showed a small effect on glucose level, which indicated that a small amount of PBA-Ins was absorbed into the bloodstream. This suggested the importance of investigating the interaction between the PBA moiety and many types of cells, such as adipocytes, in subcutaneous tissues.

## 1. Introduction

**Figure 1:**
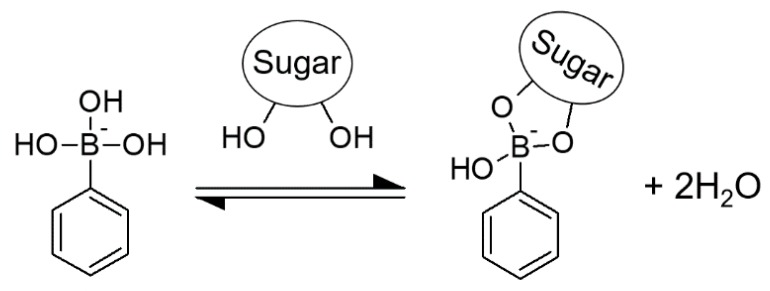
Dynamic covalent bond formation between phenylboronic acid (PBA) and sugar.

Substantial attention has been placed on phenylboronic acid (PBA) derivatives because of their unique formation of dynamic covalent bonds with the diol part of sugar molecules [1,2,3,4]. Two water molecules are removed from PBA and a diol molecule, resulting in formation of a cyclic ester (Figure 1). Although the ester linkage is a covalent bond, the formation is reversible. Therefore, this is referred to as a dynamic covalent bond, and the ester formation reaction is expressed with the concept of a chemical equilibrium.

PBA’s ability to form dynamic covalent bonds is expected to be applicable to many fields. In particular, the reaction between PBA and sugars has been widely investigated to develop blood glucose sensors [5,6,7,8,9,10,11,12] and glucose-responsive insulin delivery systems [13,14,15,16] for diabetes patients. However, the bond between PBA derivatives and glucose is not strong. For example, the binding constant between PBA and glucose is about 5 M^−1^ in a solution at physiological pH of 7.4 [17,18], which means that the dissociation constant is about 200 mM (3600 mg/dL), much higher than the normal blood glucose level. According to a criterion for diagnosing diabetes [19], a patient with 2 h plasma glucose ≥ 11.1 mM (200 mg/dL) during an oral glucose tolerance test can be diagnosed with diabetes.

Compared with glucose, *N*-acetylneuraminic acid (Neu5Ac), a type of sialic acid, shows relatively high affinity for PBA. Kataoka’s group reported that PBA’s binding constant to Neu5Ac (37.6 M^−1^) is 7.4 times higher than that for glucose (5.1 M^−1^) [18]. Neu5Ac is present on the terminal of sugar chains on the cell surface, so PBA exhibits cell adhesiveness, which has interesting applications [20,21,22,23,24]. For example, a PBA-modified polymer showed affinity for cells and enhanced cell agglutination, which resulted in the formation of spheroids [20]. In other studies, PBA-modified polymers could act as scaffolds for cells because of the affinity between PBA and cells [21,22,23]. In analytical chemistry, PBA’s affinity for sialic acids was used to develop a sensor to evaluate sialic acids’ expression levels on the surfaces of red blood cells (RBCs) [24].

**Figure 2:**
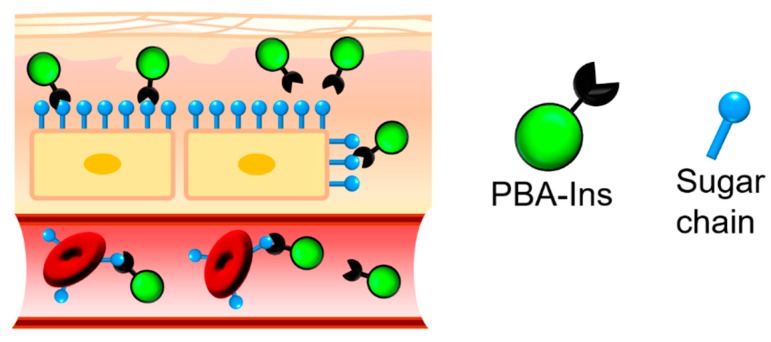
Expected interactions between PBA-modified insulin (PBA-Ins) and sugar chains on the cell surfaces in subcutaneous tissue and blood vessel.

In this study, we attempted to use PBA’s affinity for sugar chains on the cell surface to develop a new concept of a long-acting drug. We anticipated that the PBA-modified drug’s cell adhesiveness would prolong the drug activity because the cell-attached PBA-modified drug may escape from degradation and excretion, which would produce slow and long-lasting activity. We prepared PBA-modified insulin (PBA-Ins) and intravenously injected it into diabetic rats to evaluate its glucose-lowering activity duration. We also evaluated and discussed the difference between its intravenous and subcutaneous injection. Figure 2 shows the expected interactions between PBA moiety and sugar chains on the cell surface in subcutaneous tissue and blood vessels. As an in vitro study to confirm the affinity between cells and PBA-Ins, RBC agglutination experiments were also carried out.

## 2. Materials and Methods

### 2.1. Materials

Insulin (human, recombinant), sodium dodecyl sulfate, dimethylformamide, D-glucose, and D-fructose were purchased from Wako Pure Chemical Industries, Ltd. (Osaka, Japan). Tributylamine and isobutyl chloroformate were obtained from Tokyo Chemical Industry Co., Ltd. (Tokyo, Japan). 4-Carboxyphenylboronic acid, 2,4,6-trinitrobenzenesulfonic acid (TNBS, 5% in water), 4-(2-hydroxyethyl)-1-piperazineethanesulfonic acid (HEPES), and streptozotocin were purchased from Sigma-Aldrich Japan (Tokyo, Japan). A bottle of preserved sheep blood diluted with an equal volume of Alsever’s solution was obtained from Kohjin Bio Co., Ltd. (Saitama, Japan). Alsever’s solution contains 2.05% glucose, 0.8% sodium citrate, 0.055% citric acid, and 0.42% sodium chloride. The preserved blood is described here as “blood (1/2)” in accordance with the dilution ratio.

### 2.2. Preparation of PBA-Ins

In accordance with our previous report [25], PBA-Ins was prepared by condensing amino groups of insulin and 4-carboxyphenylboronic acid, using isobutyl chloroformate as a condensing agent. PBA-Ins was purified by dialysis and lyophilized. The modification ratio was evaluated with a method using TNBS [25].

### 2.3. Animal Test

Animal studies were performed in accordance with the animal use guidelines approved by the Life Science Research Center, Josai University (H28036, H29043, JU 18104 and JU 19108). Male Wistar rats (eight weeks old) were anesthetized with inhaled isoflurane, and their jugular veins were cannulated. To induce diabetes, streptozotocin was dissolved in 0.9% NaCl to achieve a concentration of 50 mg/mL, and the solution was administered at 1.0 mL/kg from the cannulated jugular vein. After 4–7 days, the rats were fasted for 20 h. Their blood glucose levels were checked, and rats exhibiting a blood glucose level higher than 250 mg/dL were used as a type 1 diabetes model. First, PBA-Ins (3 mg) was dissolved in 0.6 mL of 0.1 M NaOH aqueous solution, which was neutralized with 0.06 mL of 1 M HCl aqueous solution. The solution was diluted with Dulbecco’s phosphate-buffered saline to adjust the concentration to 190 µg/mL. Insulin (3 mg) was dissolved in 0.3 mL of 0.01 M HCl aqueous solution and diluted with Dulbecco’s phosphate-buffered saline to adjust the concentration to 190 µg/mL. The 190 μg/mL solution of PBA-Ins or insulin was administered at 1 mL/kg to the cannulated jugular vein to achieve a dose of 190 μg/kg. At a predetermined time, blood samples (100 μL) were collected from the cannulated jugular vein, and the whole blood was centrifuged at 2000× *g* for 2 min at 4 °C. Then, the glucose concentration of plasma was measured by the glucose oxidase method (Glucose CII Test Wako Kit; Wako Pure Chemical Industries, Ltd., Osaka, Japan). To investigate PBA-Ins’ prolonged effect, glucose was abdominally administered to diabetic rats 2.75 h after the first intravenous injection of PBA-Ins. Glucose was dissolved in ultrapure water to adjust the concentration to 0.5 g/mL, and the solution was abdominally administered at 2 mL/kg to achieve 1.00 g/kg. The statistical significance of differences in the results was analyzed using Student’s *t*-test (probability values less than 0.05, *p* < 0.05) for comparisons of two groups. Under the same conditions, subcutaneous injection was also performed, and the glucose level was monitored.

### 2.4. RBC Agglutination

#### 2.4.1. Investigation of an Appropriate Dilution Ratio of RBCs

Ten microliters of the preserved blood (1/2) was added to a microtube, to which 990 μL of HEPES buffer solution (200 mM, pH 7.4) containing 154 mM NaCl was also added. The sample is referred to here as blood (1/200). Fifty microliters of blood (1/200) was added to a well of a 96-well microplate (U-bottom, polystyrene, GDMP-96U; AS ONE Corporation, Osaka, Japan), and 150 μL of a HEPES buffer solution (200 mM, pH 7.4) containing 0.22 mM PBA-Ins and 154 mM NaCl was added to the well. Finally, the well contained blood (1/800) and 0.17 mM PBA-Ins (well 1). In a similar manner, the volume of the preserved blood (1/2) was adjusted to change its dilution ratio in wells 2 (blood 1/400), 3 (blood 1/320), and 4 (blood 1/160). The microplate was gently mixed and left to stand for 12 h. Then, the states of RBC agglutination were observed visually.

#### 2.4.2. The Effect of Fructose on RBC Agglutination

Ten microliters of the preserved blood (1/2) was added to a microtube, to which 990 μL of HEPES buffer (200 mM, pH 7.4) containing 308 mM fructose was also added. The sample is here referred to as blood (1/200 with fructose). Fifty microliters of blood (1/200 with fructose) was added to a well of the microplate, and 150 μL of HEPES buffer (200 mM, pH = 7.4) containing 0.22 mM PBA-Ins and 308 mM fructose was added to the well. Finally, the well contained blood (1/800), 0.17 mM PBA-Ins, and 307 mM fructose, and this procedure was repeated three times in row G of the microplate. To confirm the roles of insulin, PBA moiety, and fructose, comparative experiments were carried out in rows A, B, C, E, and F. To observe RBC agglutination with a digital microscope, the well’s content for each condition was gently mixed and sampled with a dispenser.

## 3. Results and Discussion

### 3.1. Glucose-Lowering Activity by Intravenous Injection

PBA-Ins was prepared in accordance with the previous method, and the modification number of PBA was evaluated with the TNBS method [25]. The results show that two PBA moieties were introduced into one insulin molecule. 

PBA-Ins was intravenously injected into diabetic rats treated with streptozotocin. According to the determined time, blood was sampled to monitor the blood glucose level. The PBA-Ins-administered group showed a slower decrease in the first hour and slower recovery of the glucose level than the insulin-administered group. A significant difference (*p* < 0.05) in the glucose level was observed between the PBA-Ins group and the insulin group at 8 h after injection. This result suggests that PBA modification prolonged the effect of insulin.

**Figure 3:**
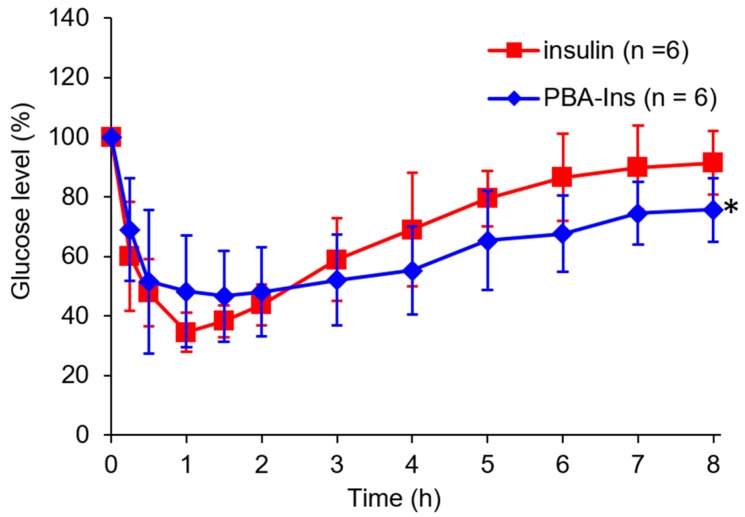
Blood glucose level profile after intravenous injection of 190 μg/kg insulin or PBA-Ins. Each value presents mean ± S.D. (*n* = 6); * means *p* < 0.05 at 8 h after intravenous administration, calculated using Student’s *t*-test.

**Figure 4:**
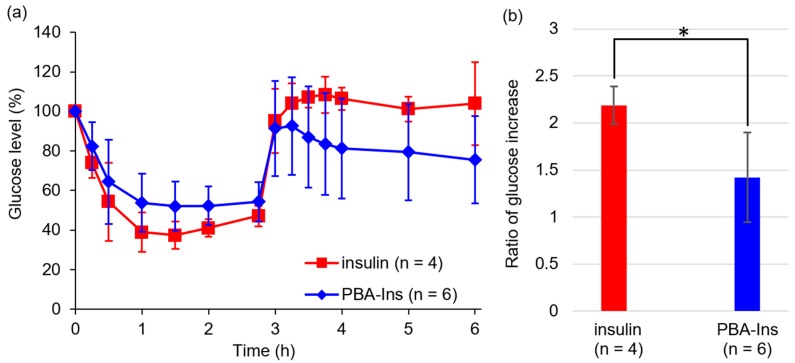
The effect of abdominal injection of glucose (1.00 g/kg) at 2.75 h after intravenous injection of insulin or PBA-Ins (190 μg/kg). (**a**) Profile of blood glucose level. Each value presents mean ± S.D. (*n* = 4 or 6). (**b**) The ratio of glucose increase, which was calculated by dividing glucose level (6 h) by glucose level (2.75 h). * means *p* < 0.05 calculated using Student’s *t*-test.

As shown in Figure 3, the glucose level did not recover rapidly in both groups of insulin and PBA-Ins, so the residence time of insulin or PBA-Ins was unclear. To investigate whether insulin or PBA-Ins was present in the body more than 3 h after the intravenous injection, glucose (1.00 g/kg) was abdominally injected at 2.75 h (Figure 4a). The ratio of the glucose level (6 h) to the glucose level (2.75 h) was calculated, and its value for PBA-Ins was compared to that of insulin (Figure 4b). The value was calculated to be 1.4 in the case of PBA-Ins, which was lower than that in the case of insulin (2.2). We considered that the lower value in the former case was because of the remaining PBA-Ins, which was regarded as further support for PBA-Ins’s prolonged effect.

### 3.2. Agglutination of RBCs

#### 3.2.1. Investigation of the Appropriate Dilution Ratio of RBCs

We proposed that the PBA-Ins’s prolonged effect was produced by the binding between PBA moiety and sugar chains on the cell surface [18,24]. In the case of intravenous injection, one of the groups of cells targeted by PBA is RBCs because there are sialic acids on the terminals of sugar chains on the RBC surfaces. In a report by Burnett et al., published in 1980, it was reported that PBA-dimer induced RBC agglutination [26]. In this study, we evaluated the PBA modification ratio by counting the remaining amino groups with the TNBS method [25]. The results showed that, on average, one PBA-Ins molecule has two PBA moieties. Thus, like the PBA-dimer reported by Burnett et al., PBA-Ins has the potential to agglutinate RBCs.

**Figure 5:**
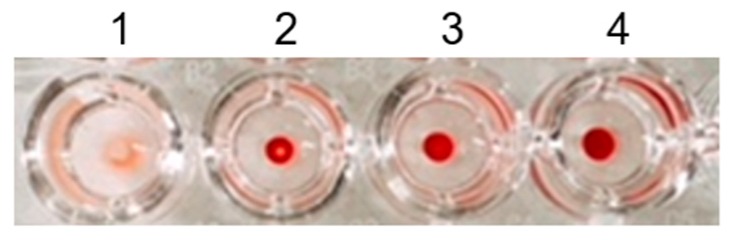
The effect of dilution ratio of blood on red blood cell (RBC) agglutination induced by PBA-Ins. Well 1 (blood 1/800), well 2 (blood 1/400), well 3 (blood 1/320), and well 4 (blood 1/160). All wells contained 0.17 mM PBA-Ins.

Figure 5 shows the effect of PBA-Ins on diluted blood samples in the microplate wells. There was no precipitation in well 1, which indicated RBC agglutination. In contrast, there was precipitation in wells 2–4, indicating no agglutination. This result confirmed that RBC agglutination was observed only in the most diluted condition (blood 1/800), which suggests that the stoichiometry between PBA-Ins and RBCs was important. The agglutination mechanism is explained as follows. When RBCs agglutinate, PBA-Ins works as a cross-linker, and a three-dimensional network is formed. To form this network, one RBC interacts with several PBA-Ins. To achieve such stoichiometry, a low concentration of RBCs and a high concentration of PBA-Ins are appropriate. However, it was difficult to prepare a PBA-Ins solution at a high concentration because of its limited solubility. We used PBA-Ins solution at 0.17 mM, and the concentration of PBA-Ins was sufficient to cross-link RBCs in well 1, the most diluted condition. However, it was not sufficient for wells 2–4.

If PBA-Ins induced RBC agglutination in blood vessels, PBA-Ins would not be suitable as a drug. However, the in vitro experiment showed that 800 times dilution was needed for PBA-Ins to induce RBC agglutination. From a stoichiometry perspective, we recognized that intravenously injected PBA-Ins does not induce RBC agglutination because RBCs were not diluted in the in vivo experiment, as shown in Figure 3 and Figure 4.

#### 3.2.2. Investigating the Role of PBA in RBC Agglutination

**Figure 6:**
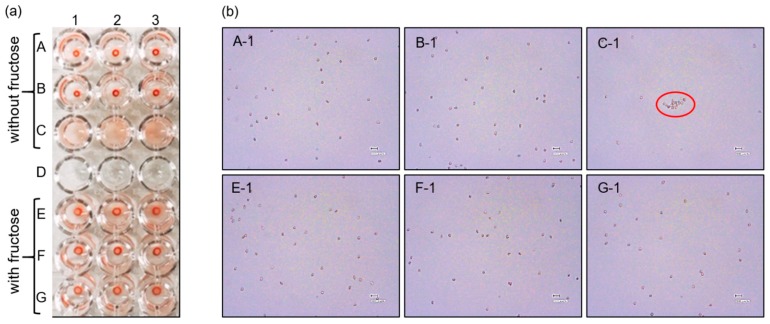
Fructose’s effect on RBC agglutination. (**a**) Visual appearance of the microplate. Each condition contained blood (1/800) and was repeated thrice in columns 1–3. The wells in rows A (without insulin or PBA-Ins), B (with insulin), and C (with PBA-Ins) lacked fructose. The wells in row E (without insulin or PBA-Ins), F (with insulin), and G (with PBA-Ins) contained 307 mM fructose. (**b**) Microphotographs of the contents of wells (column 1) for each condition. Agglutination of RBCs is marked with a red circle in the panel for C-1.

Row C of the plate in Figure 6a showed sufficient agglutination reproducibility in the condition of blood 1/800 with PBA-Ins (*n* = 3). Figure 6b shows microphotographs of the contents of column 1 for each condition. Among all conditions, RBC agglutination was found only in the condition of row C. The comparative study of row A (blood 1/800) and row B (blood 1/800 with insulin) showed no RBC agglutination, indicating that the PBA moiety is necessary for RBC agglutination.

To obtain additional evidence for the importance of the PBA moiety, fructose was used because it shows relatively high binding affinity for the PBA moiety [17]. In the presence of fructose, RBC agglutination was not observed in row G (blood 1/800 with PBA-Ins, fructose). This was probably because of a competitive inhibitory effect of fructose on the binding between PBA and sugar chains on the RBC surfaces. As another possible interpretation, fructose-bound PBA-Ins has relatively high hydrophilicity, so fructose-bound PBA-Ins does not interact with the hydrophobic cell membrane of RBCs.

The RBC agglutination experiment demonstrated PBA-Ins’s affinity for cell surfaces; however, it should be borne in mind that RBCs are only one type of cell. Not only RBCs, but also vascular endothelial cells should be considered when PBA-Ins is injected intravenously. In the case of subcutaneous injection, subcutaneous tissue cells are important; moreover, insulin analogs are usually administered subcutaneously.

### 3.3. Glucose-Lowering Activity by Subcutaneous Injection

Many diabetes patients subcutaneously self-inject insulin formulations. The injected insulin analogs are gradually absorbed from the subcutaneous tissue to capillary blood vessels. This absorption process is important for prolonged insulin formulation. Each long-acting insulin formulation has a mechanism for remaining in subcutaneous tissue for slow absorption [27,28,29]. In this study, we investigated the possibility that PBA-Ins can be used as a prolonged insulin analog via subcutaneous self-injection, because PBA-Ins may interact with subcutaneous tissue cells.

**Figure 7:**
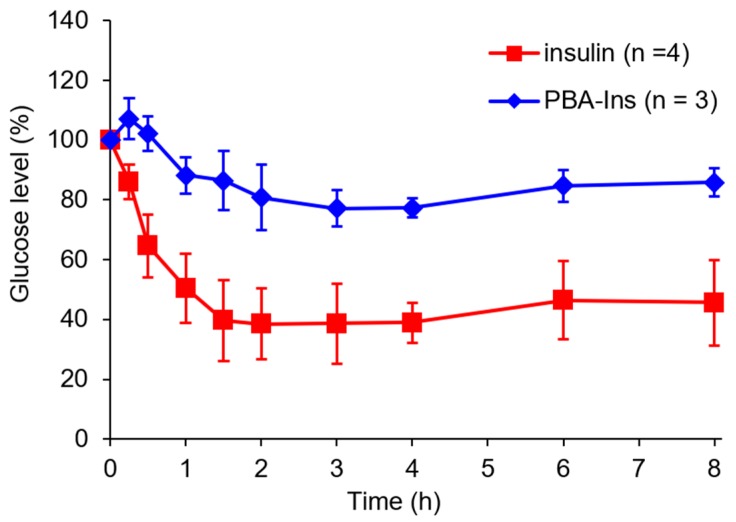
Profile of blood glucose level of subcutaneous injection of insulin or PBA-Ins (190 μg/kg). Each value presents as mean ± S.D. (*n* = 4 or 3).

PBA-Ins was subcutaneously injected, and the blood glucose level was monitored. Figure 7 shows that the glucose-lowering activity of PBA-Ins was much lower than that of native insulin. It should be noted that such a large difference between PBA-Ins and native insulin was not observed when they were intravenously administered (Figure 3).

**Figure 8:**
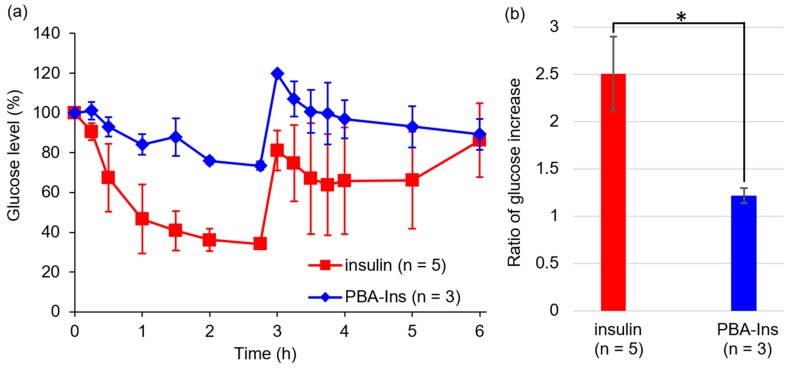
The effect of abdominal injection of glucose (1.00 g/kg) at 2.75 h after the subcutaneous injection of insulin or PBA-Ins (190 μg/kg). (**a**) Profile of blood glucose level. Each value presents mean ± S.D. (*n* = 5 or 3). (**b**) The ratio of glucose increase calculated by dividing glucose level (6 h) by glucose level (2.75 h). * means *p* < 0.05 calculated using Student’s *t*-test.

Next, we abdominally injected glucose at 2.75 h after PBA-Ins subcutaneous injection (Figure 8a). The group administered PBA-Ins showed glucose tended to decrease through 3–6 h. In contrast, the insulin-administered group showed a glucose increase during 5–6 h. To evaluate the impact of the abdominal injection of glucose on blood glucose, the ratio of glucose level (6 h) to glucose level (2.75 h) was calculated (Figure 8b). In the case of PBA-Ins, the ratio was calculated to be 1.2, which is lower than the ratio in the case of native insulin (2.5). This may suggest a prolonged effect of subcutaneously injected PBA-Ins; however, it did not exhibit marked blood glucose-lowering activity. From this result, we hypothesize that the interaction between PBA-Ins and cells in subcutaneous tissue is too strong for PBA-Ins to be absorbed into the bloodstream. In this context, a possible future challenge is to develop the effective transfer of PBA-Ins from subcutaneous tissue into capillary blood vessels by controlling the strength of interaction between PBA-Ins and cell surfaces.

## 4. Conclusions

We modified insulin with PBA and intravenously injected it into diabetic rats. Monitoring the glucose level showed that PBA-Ins has longer glucose-lowering activity than native insulin. We hypothesized that this prolonged effect of PBA-Ins was derived from the interaction between the PBA moiety and sugar chains on the cell surface. The interaction between PBA-Ins and cells was confirmed by an in vitro experiment of RBC agglutination. PBA-Ins induced RBC agglutination by working as a cross-linker for RBCs, and a competitive compound (fructose) inhibited agglutination. Unlike the intravenous injection of PBA-Ins, the subcutaneous injection of PBA-Ins showed low glucose-lowering activity. The difference of activity derived from different administration routes indicates the importance of evaluating the interaction between the PBA moiety and many types of cells, depending on the administration route. At this point, PBA-Ins is not comparable with other long-acting insulin analogues; however, this study is notable because it has provided quite a new strategy to develop long-acting drugs.
